# Branched-chain amino acids partially recover the reduced growth of pigs fed with protein-restricted diets through both central and peripheral factors

**DOI:** 10.1016/j.aninu.2021.02.002

**Published:** 2021-05-29

**Authors:** Mohammad Habibi, Cedrick Shili, Julia Sutton, Parniyan Goodarzi, Excel Rio Maylem, Leon Spicer, Adel Pezeshki

**Affiliations:** Department of Animal and Food Sciences, Oklahoma State University, Stillwater, OK, 74078, USA

**Keywords:** Branched-chain amino acid, Very low protein diet, NPY, insulin-like growth factor-I, Serotonin, Pig

## Abstract

The objective of this study was to assess the growth efficiency of pigs fed with protein-restricted diets supplemented with branched-chain amino acids (BCAA) and limiting amino acids (LAA) above the recommended levels. Following 2 weeks of adaptation, 48 young barrows were weight matched and randomly assigned to 6 treatments (8 pigs/treatment) for 4 weeks: positive control (PC) with standard protein, negative control (NC) with very low protein containing LAA (i.e., Lys, Met, Thr and Trp) at recommended levels, and NC containing LAA 25% (L25), LAA 50% (L50), LAA+BCAA (i.e., Leu, Ile and Val) 25% (LB25) and LAA+BCAA 50% (LB50) more than recommendations. Feed intake (FI) and body weight (BW) were measured daily and weekly, respectively. At week 6, blood samples were collected, all pigs euthanized and tissue samples collected. The data were analyzed by univariate GLM or mixed procedure (SPSS) and the means were separated using paired Student's *t*-test followed by Benjamini-Hochberg correction. Relative to PC, NC had decreased FI, BW, unsupplemented plasma essential amino acids, serum insulin-like growth factor-I (IGF-I) and hypothalamic neuropeptide Y (NPY) (*P* < 0.01). Compared to NC, L25 or L50, LB50 had increased BW and serum IGF-I and decreased plasma serotonin and both LB25 and LB50 had higher FI, plasma BCAA, hypothalamic 5-hydroxytryptamine-receptor 2A and NPY and jejunal 5-hydroxytryptamine-receptor 7 (*P* < 0.01). Overall, supplementation of protein-restricted diets with increased levels of dietary BCAA partially recovered the negative effects of these diets on growth through improved IGF-I concentration and FI, which was associated with changed expression of serotonin receptors, blood AA and hypothalamic NPY.

## 1 Introduction

Mineral salts, toxic metals, microorganisms, antibiotics and nitrogen (N) and its different forms such as nitrate and ammonia are among the groundwater pollutants present in animal manure (Haines and Staley, 2004). According to United States Environmental Protection Agency, the amount of excreted N from swine only, is about 24 to 103 kg per 450 kg live weight per year (Haines and Staley, 2004), which negatively impacts human and ecological health. Feeding pigs with low protein diets supplemented with individual amino acids (AA) has been suggested as a dietary strategy to reduce N excretion (Hansen et al., 1993; Shriver et al., 2003; Lordelo et al., 2008; Opapeju et al., 2009; Manjarin et al., 2012), which can contribute to the environmental health and sustainability of the swine industry. Slightly low protein (SLP) diets, i.e., diets with ≤ 4 percentage units reduced crude protein (CP) supplemented with limiting AA (LAA; i.e., Lys, Met, Thr and Trp) have been considered for weanling, growing and finishing pigs with no negative influence on the growth performance (Kerr et al., 1995, 2003; Kerr and Easter, 1995; Le Bellego et al., 2002; Shriver et al., 2003; Madrid et al., 2013; He et al., 2016). These diets not only reduce N excretion and the feed cost (Shriver et al., 2003; Lordelo et al., 2008; Opapeju et al., 2009; Manjarin et al., 2012) but also decrease the post-weaning diarrhea (Ball and Aherne, 1987; Yue and Qiao, 2008). Very low protein (VLP) diets with > 4 percentage units reduced CP may significantly reduce the N loss and environmental pollution; however, these diets limit the growth of the weaned, growing and finishing pigs (Nyachoti et al., 2006; Deng et al., 2007a; He et al., 2016). Developing novel dietary strategies that improve the growth of pigs fed with VLP diets may provide application to these diets at commercial swine production levels that will eventually contribute to environmental stewardship.

Leu, Ile, and Val are branched-chain AA (BCAA), which are involved in protein synthesis, energy homeostasis, and lipid metabolism (Zhang et al., 2017). Supplementation of a mixture of all BCAA (Zhang et al., 2013; Ren et al., 2015; Li et al., 2016; Zheng et al., 2016; Duan et al., 2016; Tian et al., 2019) or individual BCAA (Zheng et al., 2001; Figueroa et al., 2003; Lordelo et al., 2008; Nørgaard and Fernández, 2009; Yin et al., 2010; Roux et al., 2011; Zhang et al., 2016, 2018) maintains the growth of weaned and growing pigs fed with SLP diets, but there are relatively limited data on the effect of BCAA on growth of pigs fed with VLP diets. We recently showed that the degradation and endogenous biosynthesis pathways of BCAA is greatly influenced in nursery pigs fed with VLP diets (Spring et al., 2020a). Others showed that supplementation of Val to VLP diets improves the growth of growing pigs (Russell et al., 1987). Adding a mixture of a BCAA together with His and Phe to VLP diets improved growth performance of nursery pigs (Gloaguen et al., 2014). However, due to using a combination of different AA, the improved growth performance cannot be specifically attributed to BCAA in that study. We also recently demonstrated that adding BCAA at NRC (2012) levels to VLP diets partially reversed the negative effect of these diets on growth of nursery pigs (Spring et al., 2020b). It is unknown whether supplementation of BCAA together with LAA beyond NRC recommendations can completely recover the reduced growth of pigs offered with VLP diet.

Improved growth in response to BCAA has been related with enhanced muscle growth (Yin et al., 2010; Murgas Torrazza et al., 2010), intestinal cell proliferation (Sun et al., 2015; Duan et al., 2018) and feed intake (FI) (Zhang et al., 2013; Zheng et al., 2016). Addition of all BCAA (Ren et al., 2015), Val alone or combination of Val and Ile (Lordelo et al., 2008) to SLP diets increased FI in nursery pigs. Val-deficient diets significantly reduced FI (Mavromichalis et al., 1998), but combination of BCAA, His and Phe supplemented to VLP diets (Gloaguen et al., 2014) maintained FI in nursery pigs. We showed that supplementation of BCAA to VLP diets increased the FI in nursery pigs (Spring et al., 2020b). Therefore, there is evidence that supplementation of VLP diets with BCAA at NRC levels has partial stimulatory effect on FI in pigs; however, it is unknown whether adding mixture of both BCAA and LAA above NRC levels to VLP diets will completely recover the reduced FI observed with these diets.

The molecular mechanism by which FI is regulated by AA, especially by BCAA remains largely unknown in pigs. Previous research has shown that BCAA supplementation into SLP diets increases neuropeptide Y (*NPY*) and agouti-related protein (*AgRP*) (Zheng et al., 2016; Tian et al., 2019) and decreases proopiomelanocortin (*POMC*) (Tian et al., 2019), melanocortin-4-receptor (*MC4R*) (Zheng et al., 2016) and cocaine- and amphetamine-regulated transcript (*CART*) (Zheng et al., 2016) mRNA in hypothalami of nursery pigs. High dietary Leu reduces the concentration of brain Trp, which is the precursor of serotonin in weaned pigs and humans (Perez-Cruet et al., 1974; Wessels et al., 2016b) while central serotonin has been reported as an anorexigenic hormone which appears to play an important role on feeding behavior and appetite regulation in pigs (Willemen et al., 2014; Wessels et al., 2016b). Little is understood on the FI regulation in pigs fed VLP diets supplemented with BCAA. We hypothesized that supplementing BCAA along with LAA greater than NRC recommended levels could mitigate the adverse effects of VLP diets on growth via improved FI. Here for the first time, we report the effect of mixture of BCAA and LAA above NRC recommendation on FI, growth, blood metabolites, nitrogenous compounds, metabolites and hormones and gene and protein expression of markers associated with FI regulation in hypothalamus and gut in nursery pigs fed with VLP diets.

## 2 Materials and methods

### 2.1 Animals and housing

All the experimental procedures used in the current study were in accordance with Oklahoma State University Animal Care and Use Committee. The experimental protocols were approved by Oklahoma State University Animal Care and Use Committee (ACUP #AG-17-27). A total of 48 weaned barrows (Duroc sire line and Large White × Landrace dam; Seaboard, Hennessey, OK) at 3 weeks of age were used and housed in a room with controlled temperature and ventilation. The room temperature was reduced gradually from 30 °C in week 1 of study to 26 °C in wk 6 with 10-h light/14-h half-light program. Single-hole stainless steel feeders and cup waterers (Aqua Chief) with a single 1/2″ nipple (Lixit Nipple Waterer - L-70) were used. All pigs had ad libitum access to feed and water throughout the study.

### 2.2 Diets and experimental design

After an initial 2 weeks of adaptation, pigs were weight matched, individually housed and randomly assigned to 6 dietary treatments with body weight (BW) of 9.0 ± 2.9 kg (8 pigs/group) for 4 weeks: 1) positive control (PC), basal diet with 3.40 Mcal/kg ME (NRC, 2012); 2) negative control (NC), basal diet with 3.4 Mcal/kg ME containing limiting AA (LAA; i.e., Lys, Met, Thr, and Trp) at NRC (2012) recommended levels; 3) NC containing LAA 25% more than NRC (2012) recommendation (L25); 4) NC containing LAA 50% more than NRC (2012) recommendation (L50); 5) NC containing both LAA and BCAA 25% more than NRC (2012) recommendation (LB25); 6) NC containing both LAA and BCAA 50% more than NRC (2012) recommendation (LB50). Diets were formulated using National Swine Nutrition Guide (Version 2.1 Metric, ©2012 U.S. Pork Center of Excellence). To precisely meet the pigs’ nutrient requirements, phase feeding was applied. During adaptation period, all pigs were provided with nursery phase 1 (N1) diet for 1 week (d 1 to 7) and the PC of nursery phase 2 diet (N2) for an additional week (d 8 to 14). Following adaptation period, N2 diets were provided for one week (d 15 to 21), and nursery phase 3 diets (N3) were offered for 3 weeks (d 22 to 42) according to NRC recommendations. The ingredients and composition of diets are given in Table 1. The composition of AA used for diets formulations is given in Appendix Table 1. All diets were iso-caloric and all low protein diets were iso-nitrogenous. The amount of ingredients used was kept as consistent as possible across low protein diets. The energy (ME) content of all diets was kept consistent by manipulating the amount of corn and soybean meal. The level of CP among low protein diets within each feeding phase was maintained consistent (14% for N2 and 13% for N3 phases) with using different amounts of l-Ala in diets (Table 1) while the CP of PC diets for each feeding phase were based on NRC recommendations (20% for N2 and 18.6% for N3). The N1 diet was based on our previousely published study (Shili et al., 2021).

**Table 1:** Ingredients and chemical composition of experimental diets^1^ (%, as-fed basis).

Item	N2^2^						N3^3^					
	PC	NC	L25	L50	LB25	LB50	PC	NC	L25	L50	LB25	LB50
Ingredients												
Corn, yellow dent^4^	44.30	77.04	76.84	76.58	75.98	75.36	61.52	89.15	88.89	88.71	88.09	87.72
Soybean meal, 47.5% CP^4^	31.65	0.10	0.10	0.10	0.10	0.10	26.86	0.10	0.10	0.10	0.10	0.10
Fish meal, menhaden^4^	–	–	–	–	–	–	–	–	–	–	–	–
Whey, dried^4^	4.58	4.60	4.60	4.60	4.60	4.60	–	–	–	–	–	–
Corn starch^4^	14.50	6.00	6.00	6.01	6.00	6.13	7.00	0.46	0.50	0.47	0.55	0.50
Lactose^4^	–	–	–	–	–	–	–	–	–	–	–	–
Plasma spray-dried^4^	–	–	–	–	–	–	–	–	–	–	–	–
Soy protein concentrate^4^	–	–	–	–	–	–	–	–	–	–	–	–
Soybean oil^4^	–	–	–	–	–	–	–	–	–	–	–	–
Dicalcium phosphate 18.5%^4^	2.19	2.64	2.64	2.64	2.64	2.65	1.90	2.29	2.30	2.31	2.30	2.30
Limestone^4^	0.50	0.50	0.50	0.50	0.50	0.50	0.50	0.50	0.50	0.50	0.50	0.50
Salt^4^	0.50	0.50	0.50	0.50	0.50	0.50	0.50	0.50	0.50	0.50	0.50	0.50
Chromium oxide^4^	0.50	0.50	0.50	0.50	0.50	0.50	0.50	0.50	0.50	0.50	0.50	0.50
Vitamin premix^5^	0.29	0.29	0.29	0.29	0.29	0.29	0.22	0.22	0.22	0.22	0.22	0.22
Trace mineral premix^6^	0.05	0.05	0.05	0.05	0.05	0.05	0.05	0.05	0.05	0.05	0.05	0.05
SelPlex^4^	–	–	–	–	–	–	–	–	–	–	–	–
Choline chloride^4^	–	–	–	–	–	–	–	–	–	–	–	–
Zinc oxide, 72% Zn^4^	–	–	–	–	–	–	–	–	–	–	–	–
Lys, sulfate^4^	0.67	2.11	2.74	3.36	2.74	3.37	0.69	1.91	2.48	3.05	2.49	3.06
dl-Met^4^	0.12	0.27	0.37	0.47	0.37	0.47	0.10	0.23	0.32	0.41	0.32	0.41
l-Thr^4^	0.15	0.59	0.79	0.99	0.79	1.00	0.15	0.53	0.71	0.90	0.71	0.90
l-Trp^4^	0.01	0.18	0.24	0.29	0.24	0.29	0.01	0.16	0.21	0.26	0.21	0.26
l-Ile^4^	–	–	–	–	0.73	0.91	–	–	–	–	0.63	0.74
l-Val^4^	–	–	–	–	0.75	0.95	–	–	–	–	0.66	0.86
l-Leu^4^	–	–	–	–	1.13	1.51	–	–	–	–	1.01	1.38
l-Ala^4^	–	4.62	3.86	3.11	2.10	0.83	–	3.41	2.72	2.02	1.17	0.01
Calculated chemical composition^7^												
Dry matter	91.61	91.06	91.03	91.01	91.12	91.14	90.40	89.94	89.93	89.9	90.01	90.01
ME, Mcal/kg	3.40	3.40	3.40	3.40	3.40	3.40	3.36	3.36	3.36	3.36	3.36	3.36
Crude protein	20.00	14.00	14.00	14.00	14.00	14.00	18.60	13.00	13.00	13.00	13.00	13.00
Crude fiber	2.10	1.78	1.77	1.76	1.75	1.74	2.33	2.05	2.05	2.04	2.03	2.02
Crude fat	2.72	3.06	3.05	3.04	3.02	3.00	3.21	3.49	3.48	3.47	3.45	3.44
SID Lys	1.35	1.35	1.69	2.03	1.69	2.03	1.23	1.23	1.54	1.85	1.54	1.85
SID Thr	0.79	0.79	0.99	1.19	0.99	1.19	0.73	0.73	0.91	1.10	0.91	1.10
SID Met	0.39	0.39	0.49	0.59	0.49	0.59	0.36	0.36	0.45	0.54	0.45	0.54
SID Trp	0.22	0.22	0.28	0.33	0.28	0.33	0.20	0.20	0.25	0.30	0.25	0.30
SID Ile	0.74	0.21	0.21	0.21	0.93	1.11	0.67	0.22	0.22	0.22	0.84	0.95
SID Val	0.80	0.28	0.28	0.28	1.00	1.20	0.75	0.30	0.30	0.30	0.94	1.13
SID Leu	1.48	0.75	0.75	0.74	1.85	2.22	1.44	0.82	0.81	0.81	1.80	2.16
SID His	0.47	0.16	0.16	0.16	0.16	0.16	0.44	0.18	0.18	0.18	0.18	0.18
SID Arg	1.19	0.26	0.26	0.26	0.26	0.26	1.08	0.30	0.30	0.30	0.29	0.29
SID Phe	0.84	0.29	0.29	0.28	0.28	0.28	0.79	0.32	0.31	0.31	0.31	0.31
SID Phe + Tyr	1.47	0.74	0.47	0.47	0.46	0.46	1.36	0.52	0.51	0.51	0.51	0.51
Calcium	0.81	0.81	0.80	0.81	0.80	0.81	0.70	0.70	0.70	0.70	0.70	0.70
Total phosphorus	0.78	0.74	0.74	0.74	0.74	0.74	0.71	0.68	0.68	0.68	0.68	0.68
Potassium	0.92	0.35	0.35	0.35	0.35	0.35	0.78	0.30	0.30	0.30	0.30	0.30
Analyzed chemical composition												
Dry matter	87.50	88.00	87.60	87.80	88.40	88.00	87.30	86.80	87.20	87.10	86.90	87.20
Crude protein	20.00	13.60	13.80	14.50	13.70	14.00	18.00	12.40	13.40	13.10	13.20	13.20
Crude fat	1.80	2.40	2.10	2.40	2.10	2.40	2.30	2.70	2.20	2.60	2.50	2.60
Calcium	1.02	0.89	0.80	0.81	0.86	0.74	0.79	0.74	0.75	0.78	0.95	0.82
Phosphorus	0.79	0.69	0.65	0.66	0.64	0.62	0.71	0.62	0.59	0.64	0.70	0.67
Nitrogen	2.80	2.20	2.20	2.30	2.20	2.20	2.40	2.00	2.10	2.10	2.10	2.10
Ile	0.77	0.29	0.30	0.29	0.84	0.97	0.60	0.36	0.24	0.27	0.81	0.97
Val	0.81	0.30	0.29	0.30	0.98	1.17	0.62	0.42	0.24	0.29	0.77	0.93
Leu	1.45	0.80	0.68	0.72	1.71	1.92	1.25	0.84	0.66	0.73	1.82	2.10

1 PC (positive control), standard protein diet; NC (negative control), low protein diet; L25, low protein diet with supplemented limiting amino acids (LAA, i.e., Lys, Met, Thr and Trp) 25% more than NRC (2012) requirements; L50: low protein diet with supplemented LAA 50% more than NRC requirements; LB25: low protein diets with supplemented LAA and branched-chain amino acids (BCAA, i.e., Leu, Ile and Val) 25% more than NRC requirements; LB50: low protein diet with supplemented LAA and BCAA 50% more than NRC requirements.

2 PC diet was offered to all pigs from d 8 to 14 and all diets were provided from d 15 to 21.

3 Diets were provided from d 22 to 42.

4 Corn, soybean meal, fish meal, whey, corn starch, lactose, plasma spray-dried, soy protein concentrate, soybean oil, dicalcium phosphate, limestone, choline chloride, zinc oxide and salt were obtained from Nutra Blend, LLC (Neosho, MO). dl-methionine (99%) (MetAMINO) and lysine, sulfate (Biolys) were donated by Evonik (Kennesaw, GA). SelPlex was obtained from Alltech (Clovis, NM). l-threonine (98.5%) and l-tryptophan (98%) were obtained from Ajinomoto (Overland Park, KS). l-isoleucine (98.5%) was obtained from Xinjiang Fufeng group through Evonik (Kennesaw, GA). l-alanine, l-valine (96.5%) and l-leucine were obtained from Ajinomoto Health & Nutrition North America, Inc. (Raleigh, NC). Chromium oxide was purchased from Fisher Scientific (Bartlesville, OK)

5 Vitamin premix was purchased from Nutra Blend, LLC (Neosho, MO). The premix contained: vitamin A, 1,650,000 IU/kg; vitamin D_3_, 660,000 IU/kg; vitamin E, 17,600 IU/kg; vitamin K (menadione), 1,320 mg/kg; vitamin B_12_, 13.2 mg/kg; niacin, 19,800 mg/kg; D-pantothenic acid, 11,000 mg/kg; riboflavin, 3,300 mg/kg; phytase, 300,000 FYT/kg.

6 Trace mineral premix was purchased from Nutra Blend, LLC (Neosho, MO). The premix contained: copper, 11,000 mg/kg; iodine, 198 mg/kg; iron, 73,000 mg/kg; manganese, 22,000 mg/kg; selenium, 198 mg/kg; zinc, 73,000 mg/kg.

7 Values were calculated using National Swine Nutrition Guide (NSNG; Version 2.1 Metric, ©2012 U S. Pork Center of Excellence).

### 2.3 Feed intake, water intake and body weight

The single-hole feeders and calibrated buckets were used for each pen to measure the daily individual FI and water intake (WI). BW of all pigs was measured weekly. Average daily gain (ADG), average daily feed intake (ADFI), average daily protein intake (ADPI), average daily water intake (ADWI), gain-to-feed ratio (G:F), gain-to-protein ratio (G:P) and water-to-feed ratio (W:F) were calculated accordingly. Moreover, body weight gain (BWG), mean feed intake (MFI), cumulative feed intake (CFI), cumulative protein intake (CPI), G:F, and G:P were calculated on a weekly basis.

### 2.4 Feed, blood and tissue samples collection

After mixing the experimental diets for each feeding phase, feed samples were collected from different feed bags of each treatment (approximately 1 kg), pooled and stored at −20 °C until analysis. At wk 6, blood samples were collected in the ad lib fed state from all pigs via the jugular vein in the supine position into 3.0 mL lithium heparin containing tubes and 10.0 mL serum tubes (BD Vacutainer, Franklin Lakes, NJ). The collected blood samples were placed on ice, transferred to the laboratory and centrifuged at 3,000 × *g* for 15 min at 4 °C. The collected plasma and serum samples were stored at −80 °C until further analysis. At the end of study (week 6), all pigs were euthanized by CO_2_ asphyxiation method. Immediately after euthanasia the jejunum and hypothalamus samples were collected, snap-frozen in liquid nitrogen and stored at −80 °C until analysis. The hypothalamus was extracted within 5 to 10 min through dissection from midsagittal plane region of the animal's head as described previously (Shen et al., 2012) (Appendix Fig. 1).

### 2.5 Diets and supplemental amino acids composition analysis

The experimental diets and supplemented AA were analyzed for dry matter [method G-16 (oven)] (CRA, 1999), CP (method 990. 03) (AOAC, 2012c), crude fat (method 942. 16) (AOAC, 2012), crude fiber (method 978. 10) (AOAC, 2012b), and calcium and phosphorus (method 985. 01) (AOAC, 1996) and nitrogen (Gavlak et al., 2005) by Servi-Tech laboratory (Dodge City, KS; Table 1; Appendix Table 1) as we previousely described (Shili et al., 2020). Dietary AA concentration was quantified at Molecular Structure Facility, Proteomics Core of Genome Center (Davis, CA) with Na-based Hitachi 8800 according to established protocols (Cooper et al., 2001). Briefly, approximately 10 mg of feed sample was transferred into the glass hydrolysis tube (glass culture tube, VWR #47729-568) and dried under vacuum for 3 to 4 h. Then, liquid phase hydrolysis was performed in vacuo using 6 mol/L HCl and 1% phenol at 110 °C for 24 h. Next, the sample was cooled, unsealed, dried and then was dissolved in the Pickering Diluent containing 40 nmol/mL NorLeu (part #Na220). A volume of 50 μL of sample was injected and subjected to strong cation exchange to separate the AA (AminoSep Beckman Style Na+, 4 × 120 mm, part #AAA-99-6312, Concise, CA). Norleucine (CalBioChem #4890) was included as internal standard to allow correction of the results for any variations in injection volume and other chromatography variables.

### 2.6 Plasma nitrogen containing compounds analysis

Plasma nitrogen containing compounds were analyzed at Molecular Structure Facility, Proteomics Core of Genome Center (Davis, CA) with Li-based Hitachi 8900 according to established methods (Cooper et al., 2001). Briefly, plasma samples were thawed at 20 to 22 °C, acidified with 2% sulfosalicylic acid (Sigma #247006) final concentration and incubated at 20 to 22 °C for 15 min before overnight freezing the samples at −20 °C. Next day, the acidified samples were diluted with 100 nmol/mL aminoethylcysteine (AE-Cys) Li diluent (Pickering Labs, #Li220 Mountainview, CA) prior to the 50 μL injection. Free AA were separated using ion-exchange chromatography with a secondary post-column reaction with ninhydrin (WAKO, #299-70501). Column and buffers were supplied by Hitachi (Hitachi High-Technologies Corporation, Tokyo, Japan), and ninhydrin was supplied by Wako (FUJIFILM Wako Chemicals U.S.A. Corporation, Richmond, VA). Calibration of the Hitachi AA Analyzer (Model 8900, Japan) was performed using AA standards (Sigma–Aldrich, St. Louis, MO). Absorbance was recorded at both 570 and 440 nm after the reaction with ninhydrin to determine the response factor for each individual AA and to quantify levels relative to the known AA standards. The included internal standard (AE-Cys) was used to correct for any variations in injection volume caused by auto-sampler (integrated part of Hitachi 8900).

### 2.7 Plasma serotonin

Plasma serotonin concentration was measured using a commercial kit (Abcam, catalogue# ab133053) according to manufacturer's protocol. Briefly, all reagents were prepared and samples were diluted with provided phosphate buffer saline (PBS) (1 plasma:16 PBS). Then, standards and diluted samples were added to the designated wells followed by addition of serotonin antibody into the assigned wells and incubating at 20 to 22 °C for 2 h. After addition of para-Nitrophenylphosphate (pNpp) substrate into each well and incubation at 20 to 22 °C, provided stop solution was added to each well and optical density read immediately at 405 nm using Epoch microplate spectrophotometer (BioTek Instruments, Inc. Highland Park, VT). The intra-assay coefficient of variation (CV) was 4.9%.

### 2.8 Plasma glucose, triglycerides and cholesterol

After calibration of the equipment with the calibrator (Catalogue #: BL-442600, Multi-Analyte calibrator for Synchron CX/LX) the concentrations of glucose, triglycerides, and total cholesterol in plasma (>300 μL) were measured using a chemistry analyzer system (CLC 480/BioLis24i, Carolina Liquid Chemistries Corp., Brea, California) and reagents for glucose (Catalogue #: BL208), cholesterol (Catalogue #: BL211), and triglycerides (Catalogue #: BL213). The glucose absorbance was detected at 340 nm and cholesterol and triglyceride absorbance were measured at 505 nm.

### 2.9 Serum insulin-like growth factor-I

Concentration of insulin-like growth factor-I (IGF-I) in serum was determined with radioimmunoassay according to our previously published procedures (Spicer et al., 1992). A rabbit anti-human IGF-I (Catalogue #: AFP4892898) provided by the National Hormone & Peptide Program and A. F. Parlow was used as primary antibody and goat anti-rabbit IgG (H&L; Equitech-Bio, Inc. Kerrville, TX) as the secondary antibody. Briefly, IGF-I in the serum was extracted using acid-ethanol overnight. A volume of 50 μL of each extracted-neutralized sample was combined with assay buffer to make a total volume of 100 μL; primary antibody (50 μL) was added and samples were incubated for 1 h at 25 °C; 100 μL of IGF-I tracer was added and samples were incubated overnight at 4 °C; 200 μL of secondary antibody were added and incubated for 1 h at 4 °C; finally, 50 μL of the normal rabbit serum (NRS, Equitech-Bio, Inc. Kerrville, TX) were added to the samples and incubated for 1 h at 4 °C. Tubes were centrifuged at 1,800 × *g* for 25 min and supernatant was aspirated. Precipitates were counted for 2 min using an auto Gamma counter (Cobra II Auto Gamma Counter, Model D5005, American Laboratory Trading, East Lyme, CT). The intra-assay CV was 9.01%.

### 2.10 RNA isolation, reverse transcription, and quantitative PCR

RNA isolation, reverse transcription, and quantitative PCR (qPCR) were performed as we previously described (Pezeshki et al., 2012; Shili et al., 2021). Briefly, total RNA was extracted using RNeasy mini kit (Qiagen, Catalogue #: 74106, Germantown, MD) according to manufacturer's instructions and the isolated RNA quantity was determined using a NanoDrop ND-1000 spectrophotometer (Thermo Fisher, Waltham, MA). The cDNA was synthesized using the following thermocycler program (T100 Thermal Cycler, Bio-Rad, Hercules, CA): 22 °C for 5 min, 42 °C for 30 min, 85 °C for 5 min, and terminated at 4 °C. Real-time qPCR was performed (CFX96 real time PCR detection system, Bio-Rad, Hercules, CA) to quantify the mRNA abundance of target and housekeeping (β-actin) genes using specific primers (Appendix Table 2) obtained from previous studies (Nakamura et al., 2008; Smith et al., 2014; Cremer et al., 2015; Yin et al., 2015; Tian et al., 2019). The qPCR conditions were: denaturation at 50 °C for 2 min and 95 °C for 10 min, 40 cycles amplification: 95 °C for 15 s and 60 °C for 1 min, then a melt curve program: 95 °C for 15 s, 60 °C for 1 min, and 95 °C for 15 s. Finally, the 2^–ΔΔCt^ method was used for calculation of the relative mRNA abundance of target genes (Schmittgen and Livak, 2008).

### 2.11 Immunoblot analysis

Western blot was performed for MC4R, fibroblast growth factor 21 (FGF21) and tryptophan hydroxylase 1 (TPH1) in hypothalamus as we previously described (Pezeshki and Chelikani, 2014; Pezeshki et al., 2016). Briefly, frozen hypothalamus samples were ground and homogenized in a mixture of NP40 buffer (Catalogue #: FNN0021, Life Technologies, MD), protease inhibitor cocktail (Bioworld, OH) and phenylmethylsulfonyl fluoride (Catalogue #: AC215740050, Acros Organics, NJ). After homogenization (3 × 30 s) and sonication (10 s) of the samples, protein concentration was measured using Bradford assay. Following mixing of the protein homogenates with 2 X Laemmli buffer (Catalogue #: S3401, Sigma–Aldrich, MO) they were denatured at 95 °C for 3 min. The fractioning of the protein extract (40 μg; 2 mg/mL) derived from each animal was performed using 10% sodium dodecyl sulfate polyacrylamide gel electrophoresis (Catalogue #: BP166-100, Fisher Scientific, NJ). The voltage and time of gel electrophoresis was at a constant 130 V for 1 h. After separation and transferring of the proteins into the nitrocellulose membranes (100 V for 1 h 30 min), they were blocked with 5% (mass/vol) skim milk in Tris-buffered saline contained 0.1% Tween-20 (TBST-5% milk) for 1 h at 20 to 22 °C with gentle agitation. The membranes were incubated overnight at 4 °C with primary antibodies (Appendix Table 3) added to 5% skim milk (mass/vol) in TBST. Following incubation, the washed blots (for 15 min [3 × ] in TBST) were incubated with secondary antibody (Appendix Table 3) in TBST-5% milk for 1 h at 20 to 22 °C and washed in TBST (3 × 15 min). Lumi-Light Western Blotting Substrate (Novex ECL HRP Chemiluminescent Substrate Reagent Kit, Catalogue #: WP20005, Invitrogen, CA) was used for protein bands development and the images were captured using a ChemiDoc XR imaging system (Bio-Rad Laboratories Inc., CA). Densitometry was administered using Image Lab software (Version 6.0.1, Bio-Rad Laboratories Inc., CA). The glyceraldehyde 3-phosphate dehydrogenase (GAPDH; Appendix Table 3) was used as a loading control to measure the relative quantity of protein content in the samples.

### 2.12 Statistical analysis

For the overall growth, plasma glucose, triglyceride and cholesterol concentrations, serum IGF-I concentration, qPCR and immunoblotting data, outlier tests were performed followed by the analysis with GLM procedure (IBM SPSS Statistics Version 23, Armonk, NY). Blood serotonin and AA concentration data were normalized using inverse distribution function (IDF-normal). For the daily and weekly data including FI, WI, BW, BWG, MFI, CFI, CPI, G:F, and G:P the Mixed procedure was performed with consideration of the diet, time and the interaction of diet by time as fixed effects and the animal as a random variable in the model. According to the smallest levels of fit statistics for corrected Akaike Information Criterion (AIC) and Bayesian Information Criterion (BIC), the modeling of covariance structure for the repeated measurements for each variable was performed. Differences among treatment means were determined using paired Student's *t*-test followed by a Benjamini-Hochberg correction (Benjamini and Hochberg, 1995) with 0.1 false discovery rate for 6 preplanned comparisons including NC vs. PC, LB25 vs. NC, LB50 vs. NC, LB25 vs. L25, LB50 vs. L50, and LB50 vs. LB25. Differences among treatments were considered significant at *P* ≤ 0.05 and a trend at 0.05 < *P* ≤ 0.10.

## 3 Results

### 3.1 Feed and water intake and body weight

**Fig. 1:**
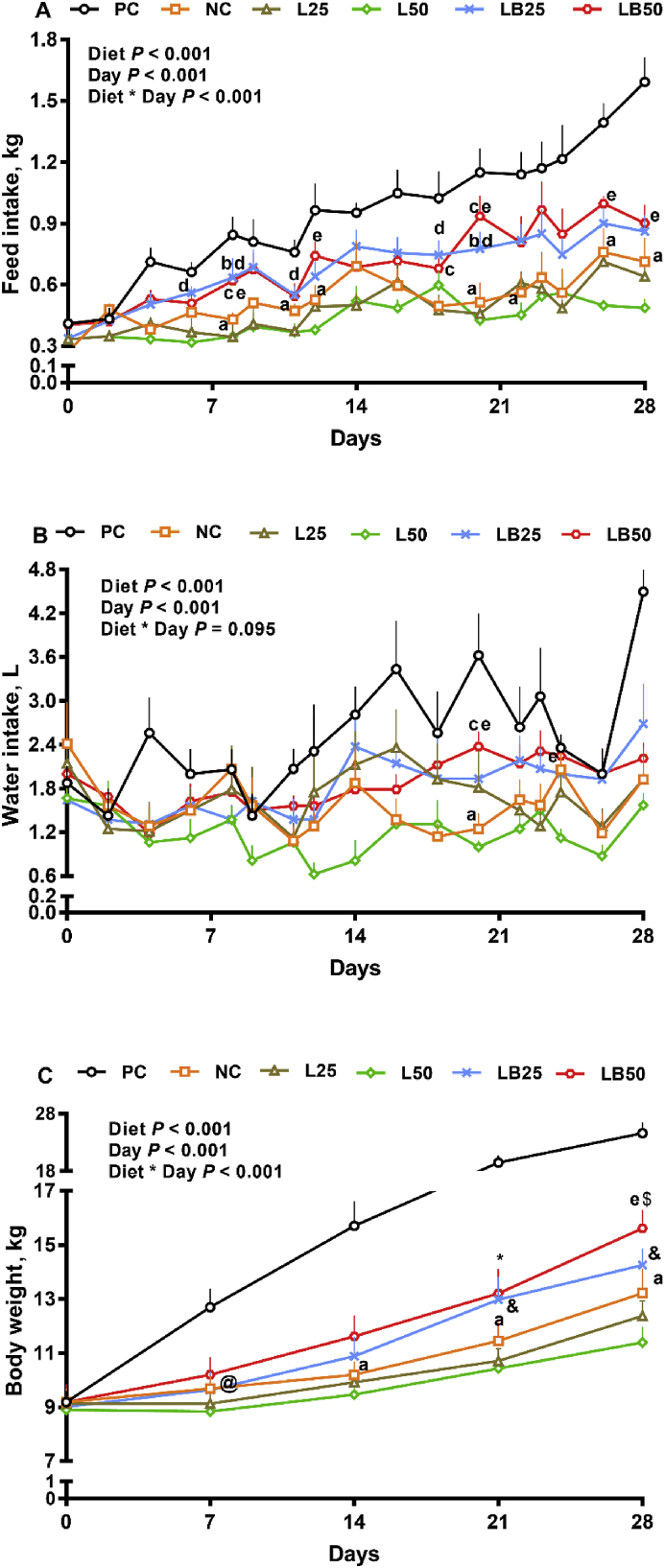
The effect of very low protein diets supplemented with combination of limiting and branched-chain amino acids on feed intake, water intake and body weight. (A) feed intake, (B) water intake, (C) body weight. PC (positive control), standard protein diet; NC (negative control), low protein diet; L25, low protein diet with supplemented limiting amino acids (LAA, i.e.*,* Lys, Met, Thr and Trp) 25% more than NRC (2012) requirements; L50: low protein diet with supplemented LAA 50% more than NRC requirements; LB25: low protein diets with supplemented LAA and branched-chain amino acids (BCAA, i.e.*,* Leu, Ile and Val) 25% more than NRC requirements; LB50: low protein diet with supplemented LAA and BCAA 50% more than NRC requirements. ^a^ *P* ≤ 0.05 NC vs. PC, ^b^*P* ≤ 0.05 LB25 vs. NC, ^c^*P* ≤ 0.05 LB50 vs. NC, ^d^*P* ≤ 0.05 LB25 vs. L25, ^e^*P* ≤ 0.05 LB50 vs. L50. ^@^*P* ≤ 0.05 NC vs. PC, ^$^*P* ≤ 0.1 LB50 vs. NC, ^&^*P* ≤ 0.1 LB25 vs. L25, ^∗^*P* ≤ 0.1 LB50 vs. L50. The values are means ± SEM, *n* = 8.

The effect of diet, day, and interaction of diet by day on daily FI was significant (Fig. 1A; *P* < 0.01). Compared to PC, i.e., basal diet with standard protein content, pigs fed with NC, i.e., VLP basal diet containing LAA (Lys, Met, Thr, and Trp) at NRC (NRC, 2012) recommendation levels, had transiently lower (37% to 55%) FI on d 8 to 28 of the study (Fig. 1A). Relative to NC, FI for LB50, i.e., NC containing both LAA and BCAA 50% more than NRC recommendation, was 39% and 82% higher on d 18 and 20, respectively (Fig. 1A; *P* < 0.01). Compared to L50, i.e., NC containing LAA 50% more than NRC recommendation, FI for LB50 tended to be higher on d 6 and it was increased by 77% to 100% on d 8, 12, 20, 26, and 28 of the experiment (Fig. 1A; *P* < 0.01). Moreover, FI tended to be higher in LB50 by 46% to 80% on d 11, 16, 23, and 24 in comparison with L50 (0.05 < *P* ≤ 0.1). LB25, i.e., NC containing both LAA and BCAA 25% more than NRC recommendation, also showed 46% and 53% higher FI than NC on d 8 and 20, respectively (Fig. 1A; *P* < 0.01). Relative to NC, LB25 tended to have greater FI on d 6, 18 and 22 by 22% to 51%. Relative to L25, i.e., NC containing LAA 25% more than NRC (NRC, 2012) recommendation, LB25 increased FI by 48% to 85% on d 6, 8, 11, 18 and 20 (Fig. 1A). No difference in daily FI was detected between LB25 and LB50. Overall, the effect of diet on ADFI, and ADPI, was significant (Table 2; *P* < 0.01). The ADFI of NC was 44.8% lower than PC (Table 2). The ADFI was 32% and 38% higher in LB25 and LB50 relative to NC, respectively. Moreover, compared to L25 and L50, ADFI was increased in LB25 and LB50 by 40% and 70%, respectively (Table 2). Compared to L50, MFI was higher in LB50 from week 1 to 4 (Table 3; *P* < 0.01). LB50 had 59.6% higher MFI than NC in week 3 but it tended to be higher in the last week. Similarly, LB25 increased MFI by 59.5% in week 2 and 43.8% in week 4 compared to L25 (Table 3). Moreover, LB25 increased MFI by 51.9% in comparison with NC at week 3 (Table 3). Compared to L50, CFI tended to be higher in LB50 during the last 3 weeks of the experiment (Table 3). Both LB25 and LB50 had higher CFI than NC in week 3 (Table 3; *P* < 0.01).

**Table 2:** Feed, protein and water intake and conversion rate in nursery pigs fed with very low protein diets supplemented with combination of limiting and branched-chain amino acids.

Item	Diets^1^						SEM	*P*-value
	PC	NC	L25	L50	LB25	LB50		
Initial BW, kg	9.18	9.18	9.13	8.90	9.01	9.18	0.19	0.99
Final BW, kg	24.56	13.22^a^	12.38	11.40	14.26^&^	15.62^$e^	0.77	<0.01
ADG, kg/d	0.53	0.12^a^	0.10	0.09	0.21^bd^	0.21^ce^	0.02	<0.01
ADFI, kg/d	0.96	0.53^a^	0.50	0.43	0.70^bd^	0.73^ce^	0.03	<0.01
ADPI, kg/d	0.18	0.07^a^	0.07	0.06	0.09^bd^	0.10^ce^	0.006	<0.01
ADWI, L/d	2.60	1.52^a^	1.49	1.18	1.73	1.85^$e^	0.09	<0.01
G:F, kg/kg	0.55	0.22^a^	0.20	0.21	0.3^bd^	0.29^$e^	0.02	<0.01
G:P, kg/kg	2.98	1.73^a^	1.49	1.58	2.31^bd^	2.18^∗^	0.10	<0.01
W:F, L/kg	2.74	3.00	3.01	2.82	2.48	2.58	0.10	0.63

SEM = standard errors of means; BW = body weight; ADG = average daily gain; ADFI = average daily feed intake; ADPI = average daily protein intake; ADWI = average daily water intake; G:F = gain-to-feed ratio; G:P = gain-to-protein ratio; W:F = water-to-feed ratio.

^a^*P* ≤ 0.05 NC vs. PC, ^b^*P* ≤ 0.05 LB25 vs. NC, ^c^*P* ≤ 0.05 LB50 vs. NC, ^d^*P* ≤ 0.05 LB25 vs. L25, ^e^*P* ≤ 0.05 LB50 vs. L50. ^@^*P* ≤ 0.1 NC vs. PC, ^$^*P* ≤ 0.1 LB50 vs. NC, ^&^*P* ≤ 0.1 LB25 vs. L25, ^∗^*P* ≤ 0.1 LB50 vs. L50. *n* = 8.

1 PC (positive control), standard protein diet; NC (negative control), low protein diet; L25, low protein diet with supplemented limiting amino acids (LAA, i.e., Lys, Met, Thr and Trp) 25% more than NRC (2012) requirements; L50: low protein diet with supplemented LAA 50% more than NRC requirements; LB25: low protein diets with supplemented LAA and branched-chain amino acids (BCAA, i.e., Leu, Ile and Val) 25% more than NRC requirements; LB50: low protein diet with supplemented LAA and BCAA 50% more than NRC requirements.

**Table 3:** Weekly body weight gain (BWG), feed intake, protein intake and feed and protein efficiency ratio of nursery pigs fed with very low protein diets supplemented with combination of limiting and branched-chain amino acids.

Item	Diets^1^^,^^2^						SEM	*P-*value
	PC	NC	L25	L50	LB25	LB50		
BWG, kg								
Wk 1	3.51	0.65^a^	0.19	0.13	0.91	1.36	0.23	<0.01
Wk 2	3.01	0.51^a^	0.91	0.52	1.40^bd^	1.55^ce^	0.17	<0.01
Wk 3	4.15	1.25^a^	1.04	0.96	2.10^bd^	1.75^e$^	0.19	<0.01
Wk 4	4.54	1.49^a^	1.42	1.10	2.04	2.07^∗^	0.21	<0.01
MFI, kg								
Wk 1	0.60	0.41^a^	0.39	0.29	0.45	0.48^e^	0.01	<0.01
Wk 2	0.84	0.54^a^	0.42	0.38	0.67^d^	0.59^e^	0.03	<0.01
Wk 3	1.05	0.52^a^	0.61	0.48	0.79^b&^	0.83^ce^	0.04	<0.01
Wk 4	1.26	0.63^a^	0.57	0.48	0.82^d^	0.81^e$^	0.04	<0.01
CFI, kg								
Wk1	4.20	2.98^a^	2.84	2.09	3.16	3.20∗	0.13	<0.01
Wk 2	5.89	3.78^a^	2.95	2.63	4.87^d#^	4.15^e^	0.23	<0.01
Wk 3	7.79	3.65^a^	4.30	3.34	5.54^b&^	6.03^ce^	0.29	<0.01
Wk 4	8.82	4.58^a^	4.03	3.51	5.75^d^	5.80^e^	0.34	<0.01
CPI, kg								
Wk 1	0.84	0.39^a^	0.38	0.30	0.44	0.47^e^	0.03	<0.01
Wk 2	1.05	0.46^a^	0.35	0.35	0.62^d#^	0.52^e^	0.04	<0.01
Wk 3	1.32	0.45^a^	0.55	0.40	0.71^b&^	0.79^ce^	0.05	<0.01
Wk 4	1.59	0.55^a^	0.53	0.44	0.75^d#^	0.75^ce^	0.06	<0.01
G:F, kg/kg								
Wk 1	0.83	0.23^a^	0.07	0.06	0.27	0.40	0.06	<0.01
Wk 2	0.52	0.14^a^	0.35	0.19	0.29^b^	0.39^c^^∗^	0.03	<0.01
Wk 3	0.53	0.34^a^	0.26	0.31	0.39	0.30	0.02	0.02
Wk 4	0.52	0.33^a^	0.36	0.32	0.36	0.37	0.02	0.06
G:P, kg/kg								
Wk 1	4.16	1.70	0.52	0.43	1.98	2.86	0.37	0.02
Wk 2	2.87	1.10^@^	2.60	1.49	2.23^b^	2.99^c^^∗^	0.20	0.02
Wk 3	2.94	2.72	1.92	2.41	2.97	2.20	0.17	0.43
Wk 4	2.88	2.70	2.67	2.47	2.72	2.78	0.14	0.98

SEM = standard error of mean; MFI = mean feed intake; CFI = cumulative feed intake; CPI = cumulative protein intake; G:F = gain-to-feed ratio; G:P = gain-to-protein ratio.

^a^*P* ≤ 0.05 NC vs. PC, ^b^*P* ≤ 0.05 LB25 vs. NC, ^c^*P* ≤ 0.05 LB50 vs. NC, ^d^*P* ≤ 0.05 LB25 vs. L25, ^e^*P* ≤ 0.05 LB50 vs. L50. ^@^*P* ≤ 0.05 NC vs. PC, ^#^*P* ≤ 0.1 LB25 vs. NC, ^$^*P* ≤ 0.1 LB50 vs. NC, ^&^*P* ≤ 0.1 LB25 vs. L25, ^∗^*P* ≤ 0.1 LB50 vs. L50. *n* = 8.

1 PC (positive control), standard protein diet; NC (negative control), low protein diet; L25, low protein diet with supplemented limiting amino acids (LAA, i.e., Lys, Met, Thr and Trp) 25% more than NRC (2012) requirements; L50: low protein diet with supplemented LAA 50% more than NRC requirements; LB25: low protein diets with supplemented LAA and branched-chain amino acids (BCAA, i.e., Leu, Ile and Val) 25% more than NRC requirements; LB50: low protein diet with supplemented LAA and BCAA 50% more than NRC requirements.

2 The *P*-values for the overall model effect for diet, week (wk) and diet × wk for BWG were <0.01, <0.01 and 0.82, for MFI were <0.01, <0.01 and < 0.01, for CFI were <0.01, <0.01 and < 0.01, for CPI were <0.01, <0.01 and < 0.01, for G:F were <0.01, 0.15 and 0.023 and for G:P were <0.01, 0.05 and 0.06, respectively.

NC had 61% lower ADPI than PC. LB25 and LB50 had 29% and 43% higher ADPI than NC, respectively. Furthermore, ADPI was 29% and 67% higher in LB25 and LB50 compared to L25 and L50, respectively (Table 2; *P* < 0.01). No differences were detected when ADFI and ADPI of LB25 were compared to those of LB50 (*P* > 0.1). CPI was also higher in LB50 than L50 from wk 1 to 4 (Table 3; *P* < 0.01). During wk 3 and 4, LB50 increased CPI by 75% and 36% compared to NC (Table 3). Relative to L25, CPI was significantly greater in LB25 during the wk 2 and 4 when LB25 also tended to have higher CPI than NC.

Overall, the effect of diet and day on WI was significant (*P* < 0.01) and the interaction of diet by day tended to be significant (Fig. 1B; *P* = 0.09). Relative to PC, WI was 65% lower on d 20 and tended to be lower (58%) on d 28 in NC (Fig. 1B). Compared to L50, WI was greater in LB50 by 137% and 100% on d 20, and 24, respectively and it was tended to be greater (150%) on d 12 (Fig. 1B). Moreover, WI for LB50 was 90% higher than that for NC on d 20 (Fig. 1B). ADWI was significantly affected (*P* < 0.01) by dietary treatments (Table 2). ADWI was 42% lower in NC than PC. LB50 increased ADWI by 22% (*P* ≤ 0.1) and 56% compared to NC and L50, respectively (Table 2). W:F ratio did not change among treatments (Table 2; *P* = 0.63).

The effect of diet, day, and the interaction of diet by day on BW was significant (Fig. 1C; *P* < 0.01). NC tended to have lower BW than PC on d 7 (0.05 < *P* ≤ 0.1) and had significantly lower BW on d 14, 21 and 28 (Fig. 1C; *P* < 0.01). BW tended to be higher (27%) in LB50 compared to L50 on d 21. Relative to L50, LB50 had 37% higher BW on d 28. LB50 tended to have higher (18%) BW than NC on d 28 (Fig. 1C; Table 2). Compared to L25, LB25 tended to have higher BW on d 21 (21%) and d 28 (15.2%) (Fig. 1C). In comparison with L25 and NC, LB25 had higher ADG. LB50 had 75% and 133% higher ADG than NC and L50, respectively (Table 2; *P* < 0.01). LB50 had higher BWG than L50 in wk 2 and 3 of the study (Table 3). Similarly, LB25 had greater BWG than L25 at wk 2 and 3 (Table 3).

Overall, the effect of diet on G:F and G:P was significant (Table 2; *P* < 0.01). Relative to NC and L25, LB25 increased G:F by 36% and 50%. Further, LB50 had 32% and 38% higher G:F than NC (*P* ≤ 0.1) and L50, respectively. Pigs fed with NC had lower G:P than those fed with PC; however, LB25 increased G:P by 33% and 55% compared to NC and L25, respectively. Moreover, G:P tended to be higher (37%) in LB50 than L50.

### 3.2 Plasma nitrogen containing compounds

The concentrations of plasma nitrogen containing compounds are shown in Table 4. Relative to PC, Lys, Ala, creatine, cystathionine, and 3-methylhistidine concentrations were increased in NC; however, Leu, Ile, Val, Phe, Arg, Tyr, Gly, Ser, Asn, Sar, βAla, L-methylhistidine and hydroxyproline (Hyp) concentrations were reduced (*P* < 0.01). In comparison with NC, Leu, Ile, Val, Gly, Ser, Sar, cystathionine, βAla, and Hyp were increased in LB25 and LB50 (*P* < 0.01). The concentrations of Met, Thr, Trp, Gln, Glu, Asp, Pro, taurine, citrulline, α-aminobutyric acid, ethanolamine, hydroxylysine, AE-cys, Orn, and l-methylhistidine did not change among low protein diets (*P* > 0.1). Compared to L25 and L50, the plasma concentrations of Leu, Ile, Val, Gly, βAla, Ser, Sar, and ammonia were increased in LB25 and LB50, respectively. The concentrations of Phe, His, creatine, and 3-methylhistidine were lower in LB25 and LB50 than L25 and L50, respectively.

**Table 4:** Plasma concentrations (nmol/mL) of nitrogen containing compounds in nursery pigs fed with very low protein diets supplemented with combination of limiting and branched-chain amino acids.

Item	Diets^1^						SEM	*P*-value
	PC	NC	L25	L50	LB25	LB50		
Lys	364.3	717.3^a^	790.7	942.0	795.4	988.1	50.8	<0.01
Met	110.9	111.6	113.3	112.8	114.6	117.4	0.9	0.39
Thr	942.5	1705.4	1781.5	2,742.0	1,590.2	2,039.8	139.4	<0.01
Trp	44.0	72.7	85.2	87.5	81.2	85.1	4.2	0.01
Leu	272.3	249.1^a^	252.5	245.0	281.9^bd^	283.0^ce^	3.3	<0.01
Ile	91.0	71.5^a^	76.1	76.5	98.1^bd^	95.9^ce^	2.1	<0.01
Val	244.1	198.6^a^	205.5	212.5	274.9^bd^	274.4^ce^	6.1	<0.01
Ala	1,360.3	1,450.1^a^	1,382.4	1,369.3	1,310.2^b&^	1,297.9^c^^∗^	13.0	<0.01
Phe	106.6	77.3^a^	80.1	73.7	31.8^bd^	60.2^cef^	4.3	<0.01
Arg	95.7	88.4^@^	91.8	86.9	81.7^bd^	82.7^c^	1.1	<0.01
His	54.3	56.5	52.9	53.1	44.9^bd^	47.9^cef^	0.8	<0.01
Tyr	87.3	73.9^a^	78.1	76.9	73.3	76.5	1.1	<0.01
Gly	1,911.5	1,288.6^a^	1,504.9	1,578.1	1,995.0^bd^	2,369.8^ce^	85.0	<0.01
Gln	967.1	681.5	637.5	591.8	818.1	678.6	31.3	<0.01
Glu	275.4	250.9^@^	241.7	253.2	259.7	261.0	2.8	0.01
Ser	333.6	324.0^@^	324.7	317.2	348.6^bd^	341.1^ce^	2.4	<0.01
Asp	31.2	29.2	28.2	28.1	28.9	28.9	0.3	<0.01
Asn	128.7	114.2^a^	115.1	112.5	121.0^b&^	117.5	1.2	<0.01
Pro	511.6	404.8	389.4	355.5	354.6	375.6	13.8	<0.01
Taurine	119.8	118.6	118.9	121.7	115.2	117.5	1.1	0.68
Sar	78.4	57.6^a^	55.3	55.8	67.2^bd^	81.3^cef^	2.3	<0.01
Creatine	133.1	151.4^a^	154.6	159.0	139.7^bd^	140.4^ce^	1.9	<0.01
Citrulline	91.0	108.6	120.1	100.7	92.8	100.1	3.3	0.11
Abu	26.9	32.0	50.6	56.4	48.4	43.8	2.9	0.01
Cystathionine	2.8	3.6^@^	4.1	4.7	5.7^b&^	5.6^c^	0.3	<0.01
βAla	22.3	20.1^a^	21.1	21.2	23.1^bd^	24.3^cef^	0.3	<0.01
Ethanolamine	14.3	14.6	16.8	21.1	17.0	22.5	1.0	0.09
Hyl	13.3	12.3	13.1	13.8	14.6	15.6	0.7	0.77
AE-Cys	139.3	137.6	140.2	141.6	140.3	144.4	0.9	0.46
Orn	133.4	128.0	128.3	125.1	123.4	128.4	0.9	0.05
l-Methylhistidine	24.41	21.63^@^	20.14	19.30	21.19	21.01	0.4	<0.01
3-Methylhistidine	6.3	10.6^a^	8.3	8.7	4.6^bd^	4.9^ce^	0.5	<0.01
Hyp	191.5	103.7^a^	116.2	100.2	134.5^b^	150.5^ce^	6.4	<0.01
Ammonia	322.4	316.6	314.5	326.3	346.8^#d^	346.7^ce^	3.6	<0.01

SEM = standard errors of means; Abu = α-aminobutyric acid; AE-Cys = S-β-aminoethylcysteine; Hyl = hydroxylysine; Hyp = hydroxyproline.

^a^*P* ≤ 0.05 NC vs. PC, ^b^*P* ≤ 0.05 LB25 vs. NC, ^c^*P* ≤ 0.05 LB50 vs. NC, ^d^*P* ≤ 0.05 LB25 vs. L25, ^e^*P* ≤ 0.05 LB50 vs. L50, ^f^*P* ≤ 0.05 LB50 vs. LB25. ^@^*P* ≤ 0.1 NC vs. PC, ^#^*P* ≤ 0.1 LB25 vs. NC, ^&^*P* ≤ 0.1 LB25 vs. L25, ^∗^*P* ≤ 0.1 LB50 vs. L50. *n* = 6.

1 PC (positive control), standard protein diet; NC (negative control), low protein diet; L25, low protein diet with supplemented limiting amino acids (LAA, *i.e.,* Lys, Met, Thr and Trp) 25% more than NRC (2012) requirements; L50: low protein diet with supplemented LAA 50% more than NRC requirements; LB25: low protein diets with supplemented LAA and branched-chain amino acids (BCAA, *i.e.,* Leu, Ile and Val) 25% more than NRC requirements; LB50: low protein diet with supplemented LAA and BCAA 50% more than NRC requirements.

### 3.3 Blood serotonin, insulin-like growth factor-I, glucose, triglycerides and cholesterol

**Fig. 2:**
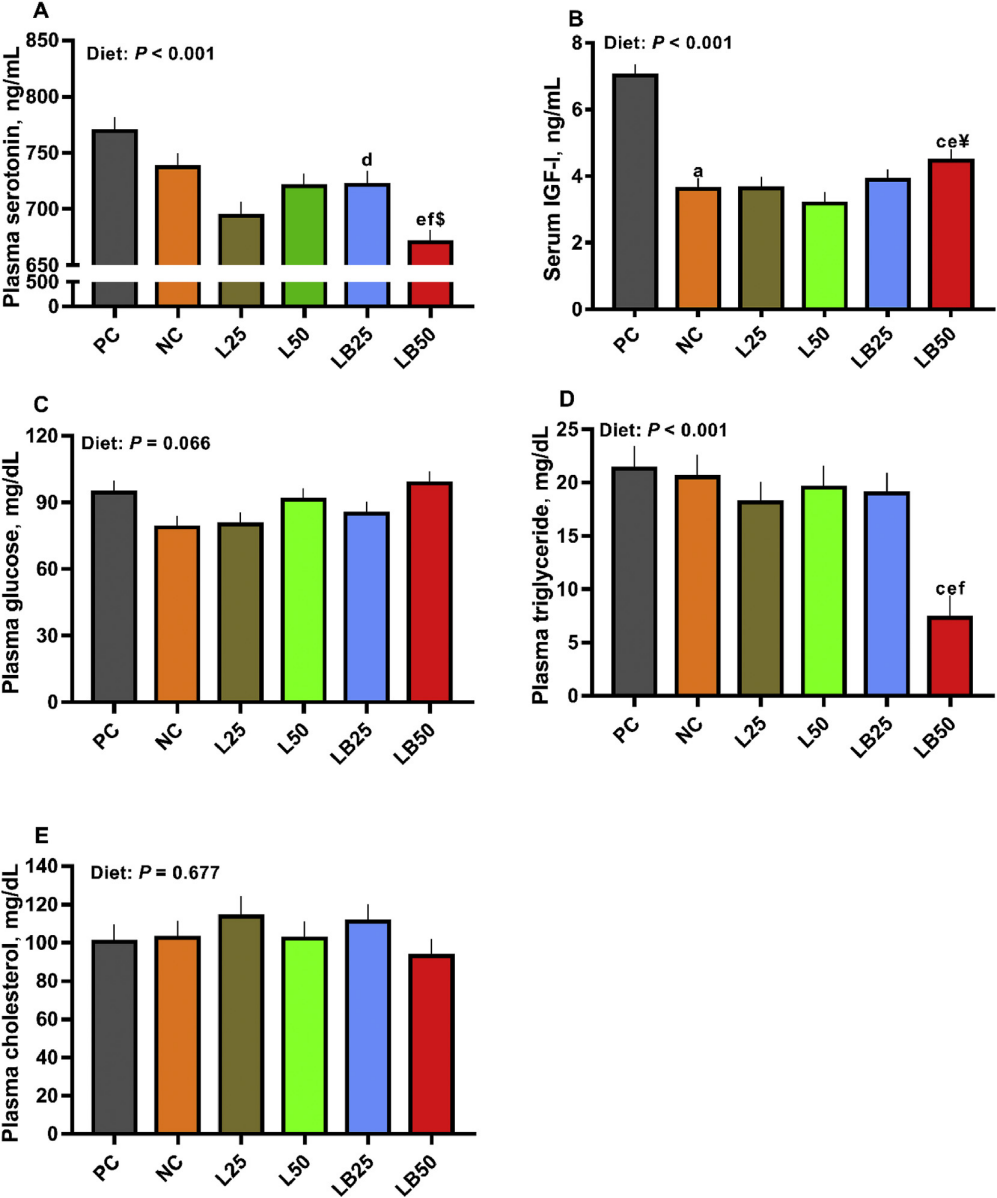
The effect of very low protein diet supplemented with combination of limiting and branched-chain amino acids on blood hormones and metabolites. (A) plasma serotonin, (B) serum insulin-like growth factor 1 (IGF-I), (C) plasma glucose, (D) plasma triglyceride, (E) plasma cholesterol. PC (positive control): standard protein diet; NC (negative control): low protein diet; L25: low protein diet with supplemented limiting amino acids LAA, i.e., Lys, Met, Thr and Trp 25% more than NRC (NRC, 2012) requirements; L50: low protein diet with supplemented LAA 50% more than NRC requirements; LB25: low protein diets with supplemented LAA and branched-chain amino acids (BCAA, i.e., Leu, Ile and Val) 25% more than NRC requirements; LB50: low protein diet with supplemented LAA and BCAA 50% more than NRC requirements. ^c^*P* ≤ 0.05 LB50 vs. NC, ^d^*P* ≤ 0.05 LB25 vs. L25, ^e^*P* ≤ 0.05 LB50 vs. L50, ^f^*P* ≤ 0.05 LB50 vs. LB25. ^$^*P* ≤ 0.1 LB50 vs. NC, ^¥^*P* ≤ 0.1 LB50 vs. LB25. The values are means ± SEM, *n* = 8.

The effect of diet on plasma serotonin concentration was significant (Fig. 2A; *P* < 0.01). Relative to NC, the plasma serotonin concentration tended to be lower in LB50 (Fig. 2A). Plasma serotonin concentration was reduced by 7% in LB50 compared to L50 (Fig. 2A) and was increased by 4% in LB25 relative to L25 (Fig. 2A). Compared to LB25, plasma serotonin concentration was decreased in LB50 by 7%.

The effect of diet on serum IGF-I concentration was significant (Fig. 2B; *P* < 0.01). The concentration of serum IGF-I was reduced by 48% in NC relative to PC and IGF-I concentration increased by 24% in LB50 compared to NC (Fig. 2B). Compared to L50, LB50 increased serum IGF-I by 40% and serum IGF-I tended to be greater (15%) in LB50 compared to LB25 (Fig. 2B).

The effect of diet on plasma glucose concentration tended to be significant, with the highest concentrations for PC, L50 and LB50 (Fig. 2C; *P* = 0.06). When pairwise comparisons were performed, no significant differences were detected (Fig. 2C). Plasma triglyceride concentration was significantly affected by dietary treatments (*P* < 0.01). Plasma triglyceride was lower in LB50 by 64%, 62%, and 61% than NC, L50, and LB25, respectively (Fig. 2D). Dietary treatment effect on plasma cholesterol concentration was not significant (Fig. 2E; *P* = 0.67).

### 3.4 The mRNA abundance of key molecules of feed intake regulation in hypothalamus and jejunum

**Fig. 3:**
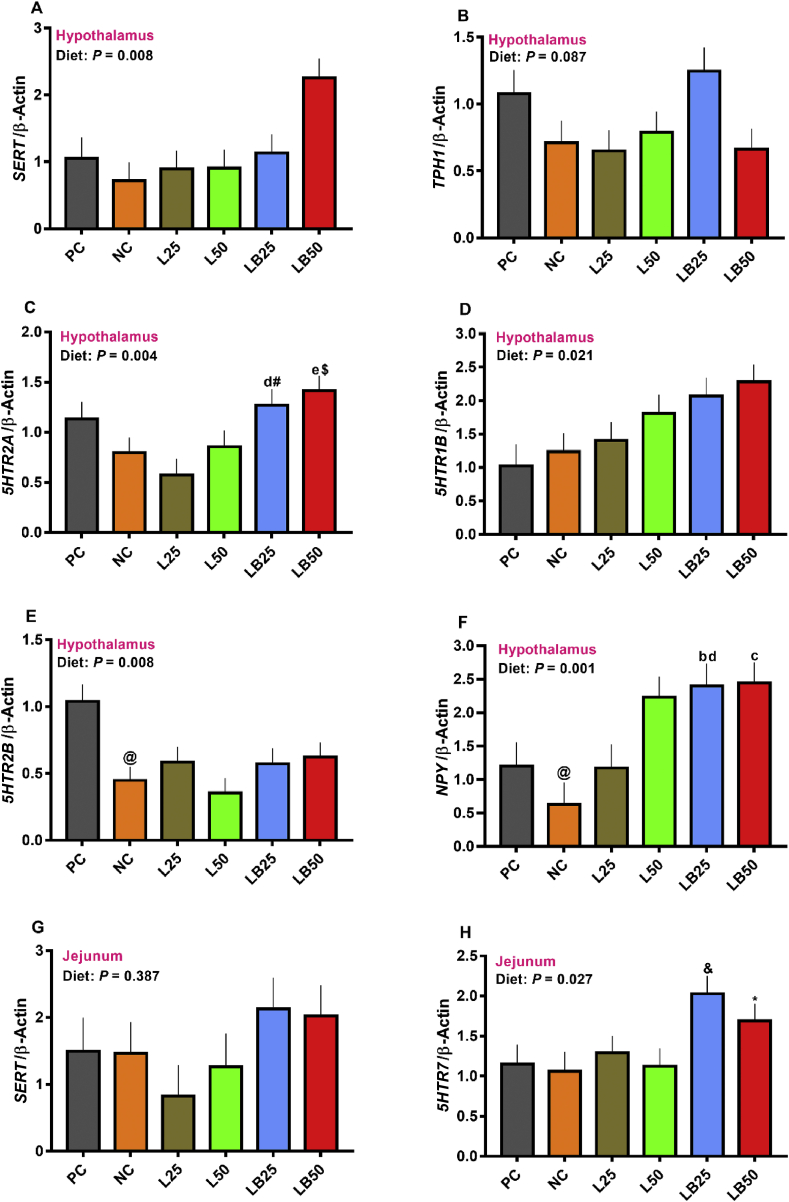
The effect of very low protein diets supplemented with combination of limiting and branched-chain amino acids on mRNA abundance of feed intake markers in hypothalamus and jejunum. (A) serotonin transporter (*SERT*), (B) tryptophan hydroxylase 1 (*TPH1*), (C) 5-hydroxytryptamin-R2A (*5HTR2A*), (D) 5-hydroxytryptamin-R1B (*5HTR1B*), (E) 5-hydroxytryptamin-R2B (*5HTR2B*), (F) neuropeptide Y (*NPY*), (G) serotonin transporter (*SERT*), (H) 5-hydroxytryptamin-R7 (*5HTR7*). PC (positive control), standard protein diet; NC (negative control), low protein diet; L25, low protein diet with supplemented limiting amino acids (LAA, i.e*.,* Lys, Met, Thr and Trp) 25% more than NRC (NRC, 2012) requirements; L50: low protein diet with supplemented LAA 50% more than NRC requirements; LB25: low protein diets with supplemented LAA and branched-chain amino acids (BCAA, i.e*.,* Leu, Ile and Val) 25% more than NRC requirements; LB50: low protein diet with supplemented LAA and BCAA 50% more than NRC requirements. The relative mRNA abundance was determined by qPCR with using β-actin as a reference target. ^b^*P* ≤ 0.05 LB25 vs. NC, ^c^*P* ≤ 0.05 LB50 vs. NC, ^d^*P* ≤ 0.05 LB25 vs. L25, ^e^*P* ≤ 0.05 LB50 vs. L50. ^@^*P* ≤ 0.05 NC vs. PC, ^#^*P* ≤ 0.1 LB25 vs. NC, ^$^*P* ≤ 0.1 LB50 vs. NC, ^&^*P* ≤ 0.1 LB25 vs. L25, ^∗^*P* ≤ 0.1 LB50 vs. L50. The values are means ± SEM, *n* = 8.

The effect of diet on mRNA abundance of hypothalamic serotonin transporter (*SERT*) and 5-hydroxytryptamine-receptor 1B (*5HTR1B*) was significant with the greatest abundance for LB50 and LB25 (Fig. 3A and D; *P* < 0.05). The mRNA expression of hypothalamic *TPH1* tended to be significant (Fig. 3B; *P* = 0.08). The transcript of hypothalamic 5-hydroxytryptamine-receptor 2A (*5HTR2A*) was greater in LB25 and LB50 compared to L25 and L50, respectively and it tended to be greater in LB25 and LB50 in comparison with NC (Fig. 3C; *P* < 0.01). The mRNA abundance of hypothalamic 5-hydroxytryptamine-receptor 2B (*5HTR2B*) tended to be lower for NC compared to that in PC group, but no differences were detected among low protein diets (Fig. 3E). The mRNA expression of hypothalamic *NPY* tended to be lower in NC than PC (Fig. 3F). The mRNA abundance of hypothalamic *NPY* was higher in LB25 compared to NC and L25 and that was greater for LB50 relative to NC (Fig. 3F; *P* < 0.01). The mRNA abundance of jejunal *SERT* did not change across diets (Fig. 3G; *P* = 0.38). The overall effect of diet on transcript of jejunal 5-hydroxytryptamine-receptor 7 (*5HTR7*) was significant and it tended to be greater in LB25 and LB50 compared to L25 and L50, respectively (Fig. 3H; *P* = 0.02). No differences among treatments were detected for the mRNA abundance of hypothalamic *5HTR7* and *POMC* and jejunal *TPH1*, *5HTR2B*, cholecystokinin (*CCK*), glucagon (*GCG*), peptide YY (*PYY*) and taste receptor type 1 member 1 (T1R1) (Appendix Fig. 2A–H).

### 3.5 The protein abundance of key molecules of feed intake regulation in hypothalamus

**Fig. 4:**
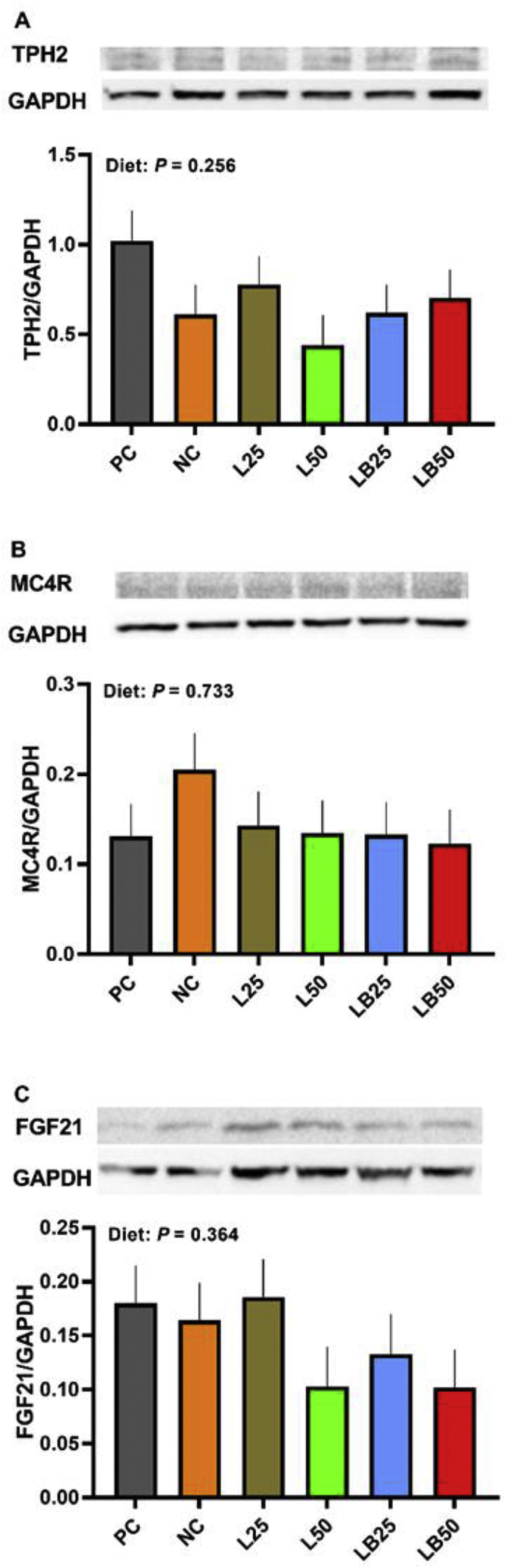
The effect of low protein diet supplemented with combination of limiting and branched-chain amino acids on relative protein abundance of feed intake markers in hypothalamus. (A) Tryptophan hydroxylase 2 (TPH2), (B) melanocortin-4-receptor (MC4R), (C) fibroblast growth factor 21 (FGF21). PC (positive control), standard protein diet; NC (negative control), low protein diet; L25, low protein diet with supplemented limiting amino acids (LAA, i.e.*,* Lys, Met, Thr and Trp) 25% more than NRC (NRC, 2012) requirements; L50: low protein diet with supplemented LAA 50% more than NRC requirements; LB25: low protein diets with supplemented LAA and branched-chain amino acids (BCAA, i.e.*,* Leu, Ile and Val) 25% more than NRC requirements; LB50: low protein diet with supplemented LAA and BCAA 50% more than NRC requirements. The relative protein abundance was determined by immunoblot analysis with using glyceraldehyde 3-phosphate dehydrogenase (GAPDH) as a reference target. Samples are derived from the same experiment and blots were processed in parallel. The values are means ± SEM, *n* = 8.

The relative protein abundance of TPH2 (*P* = 0.25), MC4R (*P* = 0.73), and FGF21 (*P* = 0.36) was not different among dietary treatments (Fig. 4A–C).

## 4 Discussion

Excessive N excretion from swine production is a threatening factor not only for the environment (Haines and Staley, 2004), but also for sustainability of the swine industry. Unlike SLP diets supplemented with LAA that have no negative effects on the growth performance of pigs (Kerr et al., 1995, 2003; Kerr and Easter, 1995; Le Bellego et al., 2002; Shriver et al., 2003; Madrid et al., 2013; He et al., 2016), VLP diets negatively impact the growth in spite of the supplementation of LAA (Nyachoti et al., 2006; Deng et al., 2007a; Yue and Qiao, 2008; He et al., 2016). We recently demonstrated that the metabolism of BCAA is greatly influenced when pigs are fed with VLP diets (Spring et al., 2020a) and in a follow up study showed that supplementing VLP diets with BCAA at NRC (NRC, 2012) levels partly reversed the negative effect of these diets on growth performance of pigs (Spring et al., 2020b). It is unknown whether supplementation of BCAA together with LAA higher than NRC recommendations can fully recover the reduced growth of pigs fed with VLP diets and whether this improvement occurs through regulation of markers associated with FI control. The objective of this study was to investigate the effect of BCAA and LAA supplemented to VLP diets at 25% and 50% higher than NRC (NRC, 2012) recommendations on growth, FI, blood metabolites, nitrogenous compounds, hormones and metabolites and gene and protein expression of markers associated with FI regulation in nursery pigs. As expected VLP diets supplemented with LAA at NRC levels without added BCAA (i.e., NC) reduced the growth-related measures such as FI and BW, serum IGF-I and expression of hypothalamic *NPY*. Our study generated several important findings: 1) diets supplemented with BCAA + LAA 25% (i.e., LB25) or 50% (i.e., LB50) more than NRC recommendation, but not those over supplemented with LAA (i.e., L25 and L50), improved the ADFI, ADPI, ADG, BW, G:F and G:P compared to NC, 2) LB50 decreased plasma serotonin and triglyceride concentrations relative to L50 and LB25, 3) LB50 increased the serum IGF-I compared to NC and L50, 4) LB25 and LB50 increased plasma concentrations of Leu, Ile, Val, Gly, Ser, Sar, and βAla relative to NC suggestive of an improved blood AA profile in these groups, 5) the mRNA abundance of hypothalamic *5HTR2A* and jejunal *5HTR7* were increased in LB25 and LB50, but not in L25 and L50, compared to L25 and L50, respectively. Further, LB25 and LB50 increased the mRNA abundance of hypothalamic *NPY* relative to NC. Overall, compared to NC, VLP diets with increased levels of dietary BCAA increased growth possibly through improved IGF-I concentration, FI and feed efficiency, which was associated with changes in serotonin concentration and its receptors gene expression, blood AA and hypothalamic NPY abundance.

To our knowledge there is no published study on the effect of supplemental BCAA higher than NRC levels on growth of pigs fed with VLP diets. Like previous studies (He et al., 2016; Peng et al., 2016; Spring et al., 2020a), VLP diet decreased the BW and growth of pigs in this study, which was partly recovered by supplementation of BCAA at 25% or 50% above NRC recommendation, but not with over supplementation of LAA only. Similar to outcome of over supplemented BCAA groups in our study, others showed that when all 3 BCAA (Yin et al., 2020), individual BCAA (Russell et al., 1987) or mixture of BCAA with other essential AA (Gloaguen et al., 2014) supplemented to VLP diets at recommended levels (NRC, 2012), the BW is partially recovered in nursery pigs. Two other studies that considered the BCAA supplementation to VLP diets at NRC levels in pigs did not include a negative control (i.e., VLP without supplemented BCAA) in their experimental design in order to compare the outcome of BCAA supplementation (Deng et al., 2009; Peng et al., 2016). In another study, a 2-fold dose BCAA supplementation into SLP diet did not further improve weanling piglets’ growth and BW compared to the ones with supplemented BCAA at positive control level (Zheng et al., 2016). Others showed that supplementation of SLP diets with BCAA at 150% of levels suggested by NRC failed to promote the growth of nursery pigs (Tian et al., 2019). The VLP diets in our study, as expected were deficient in other important AA such as His, Arg and Phe. Whether these AA are limiting for VLP diets and their supplementation along with other LAA can fully recover the growth of pigs fed with VLP diets needs further investigation. The lack of difference in growth or better performance by groups over supplemented with BCAA vs. ones receiving the recommended levels of BCAA might be due to greater AA imbalance in the latter mentioned groups.

The impaired growth in pigs fed with VLP diet may be due to reduced FI, which was partly recovered following BCAA supplementation at 25% or 50% more than recommended levels in the current study. The reduced FI in VLP diet is in line with our previous study (Spring et al., 2020a). Others showed that adding BCAA at or 100% above the required levels to SLP diet recovered the FI in weanling pigs (Zhang et al., 2013; Ren et al., 2015; Zheng et al., 2016). In another study, over supplementation of SLP diets with BCAA by 150% did not improve the FI, but adequate supplementation of BCAA increased the ADFI of nursery pigs in the same study (Tian et al., 2019). Similar to results of current study, we (Spring et al., 2020b) and others showed that when BCAA is supplemented to VLP diets at NRC levels, the FI is partially restored (Yin et al., 2020). These data suggest that BCAA supplementation at NRC level improves the FI of pigs fed with VLP diets, but over supplementation of BCAA does not further increase the FI likely due to plasma AA imbalance. Along with reduced FI, lower concentration of serum IGF-I in pigs fed with VLP diets may contribute to impaired growth in these animals. IGF-I has long been considered as a key molecule for nutritional control of growth (Straus, 1994). Similar to our data, others showed that protein restriction decreased blood IGF-I concentration (Deng et al., 2007b; Wan et al., 2017). Supplementation of BCAA at 50% above the NRC levels partly restored the reduced IGF-I concentration in pigs fed with VLP diets in the current study, which may contribute to improved growth in these animals. Not only the amount of ingested protein but also the essential AA content of diet is important regulator of IGF-I (Thissen et al., 1994). In support of our data, other studies showed that feeding BCAA or Leu metabolite increased the IGF axis activity in pigs (Buddington et al., 2018), rats (Zheng et al., 2009; Kovarik et al., 2010; Gerlinger-Romero et al., 2011) and humans (Li et al., 2015). Nevertheless, the addition of LAA and BCAA more than recommended levels failed to fully recover the reduced FI and blood IGF-I in pigs fed with VLP diet, which could be partly due to the deficiency of other essential AA such as Arg, His, or Phe in VLP diets. Although it appears that BCAA induced growth is associated with increased blood IGF-I concentration, further research is required to better understand the underlying mechanisms by which BCAA regulate the IGF axis.

Data on the effect of supplemental BCAA on FI regulatory markers in pigs fed with protein restricted diets are scarce. In this study, for the first time we showed that pigs fed with VLP diet had a lower hypothalamic NPY transcript, but supplementing this diet with BCAA above the recommended levels increased the mRNA abundance of hypothalamic *NPY*. Others reported an increased mRNA abundance of *NPY* and *AgRP* in hypothalamus of nursery pigs fed with SLP diets supplemented with BCAA (Zheng et al., 2016; Tian et al., 2019); however, adding Val alone to SLP diet failed to increase the expression of hypothalamic *NPY* (Zhang et al., 2018). Unlike our study, where we reported no effect of supplemental BCAA on hypothalamic POMC and MC4R in VLP fed pigs, others showed a decreased expression of these molecules in hypothalamus when SLP diets were supplemented with BCAA (Zheng et al., 2016; Tian et al., 2019) or Val (Zhang et al., 2018) in pigs. Further, intestinal transcript of *CCK*, *GCG*, *PYY* and *T1R1* were not affected by dietary treatments in our study, whereas others reported a decreased mRNA abundance of intestinal *T1R1*, *CCK* and *GCG* following BCAA supplementation (Tian et al., 2019) and a reduced *CCK* in the stomach, but not in the duodenum, after Val supplementation (Zhang et al., 2018) in pigs fed with SLP diets. The inconsistency in expression of these markers among studies might be attributed to the level of dietary protein and ratio of supplemental BCAA to other essential AA. In our study, unlike others (Zheng et al., 2016; Tian et al., 2019) where the ratio of BCAA to other essential AA was changed in over supplemented BCAA groups, the ratio of LAA and BCAA were kept consistent in both LB25 and LB50 groups. NPY and AgRP have stimulatory effect on FI, whereas POMC, MC4R, PYY and CCK have inhibitory effect on FI (Morton et al., 2006; D'Alessio, 2008). Increased hypothalamic *NPY* expression may contribute to increased FI in BCAA supplemented group in the present study.

Serotonin and its receptors in the gut and hypothalamus were assessed as another FI regulatory pathway in the current study. Supplementing VLP diets with BCAA at 50% more than NRC requirements decreased the plasma serotonin concentration. Reduced plasma serotonin concentration in LB50 group is in parallel with a previous report where excessive Leu supply reduced the blood serotonin concentration (Wessels et al., 2016a). The neurotransmitter serotonin (5-hydroxytryptamine, 5-HT) is a known FI regulator which is largely produced in the gastrointestinal tract (> 95%) and only 5% of body serotonin is synthesized in the brain (Lam et al., 2010; Willemen et al., 2014; Yabut et al., 2019). The reduction in blood serotonin is likely due to the influence of BCAA in blocking Trp uptake into enterochromaffin cells in the gut, which serves as a serotonin synthesis precursor (Millet et al., 2015). Despite the fact that serotonin is mostly produced in the gut and systemic administration of serotonin results in a normal satiety behavior in rats (Edwards and Stevens, 1991), the effect of peripheral serotonin on FI regulation is not completely known and thus, further research is required to understand the mechanisms by which peripheral serotonin is involved in appetite control. Limited data are available on the relationship between dietary BCAA and serotonin receptors in periphery and the brain and the role of these receptors in energy balance modulations by BCAA. Adding BCAA 50% above the required levels to VLP diet increased the mRNA abundance of hypothalamic *5HTR2A* and jejunal *5HTR7*. Given the role of central 5HTR2A in normal feeding behavior (Gorwood et al., 2018) and peripheral 5HTR7 in energy balance control (Crane et al., 2015), further studies are warranted to investigate the effect of dietary BCAA on regulation of these receptors and their role in mediating the effect of BCAA on FI regulation.

The plasma concentrations of Leu, Ile, Val, Phe, Arg, Tyr, Gly, Asn, Sar, βAla, and Hyp concentrations were reduced in pigs fed with VLP diet in the present study. This is in line with our previous study and others where it was shown that the circulating concentrations of BCAA along with other unsupplemented AA are decreased and the metabolism of AA, especially BCAA is largely influenced in protein-restricted diets (Figueroa et al., 2003; Zhang et al., 2013; Ren et al., 2015; Tian et al., 2019; Spring et al., 2020a). Reduced FI in pigs fed with VLP diet may be due to alterations in blood AA composition that result in AA imbalance sensed by hypothalamus (Wu, 2009; Zhang et al., 2013). Supplementing VLP diets with BCAA at 25% and 50% above the required levels increased the plasma concentrations of Leu, Ile, Val, Gly, Ser, Sar and βAla. This is in agreement with a previous study that showed supplementation of BCAA into protein restricted diets restored plasma pool of BCAA and Ser (Tian et al., 2019). The BCAA as essential AA are involved in FI regulation and BCAA-deficient diets have been reported to inhibit FI in piglets (Zhang et al., 2013; Zheng et al., 2016). The improved BCAA profile in circulation may contribute to increased FI and growth in LB25 and LB50 compared to NC in our study. Further research is needed to fully understand other parallel mechanisms involved in FI regulation when diets are over supplemented with BCAA. In our study, although none of experimental diets fully recovered the negative effect of VLP diets on growth, greater growth performance, serum IGF-I concentration and hypothalamic expression of *NPY* were seen in pigs fed with LB50 compared to NC, which is suggestive of the potential of BCAA in annulling the negative outcomes of VLP diets on growth. Our study has shed light on the importance of BCAA as LAA for VLP diets, but whether supplementation of BCAA at levels higher than NRC levels can be considered a cost-effective strategy to be applied in commercial swine production requires further studies to determine the optimal combination of individual BCAA to be supplemented in VLP diets.

## 5 Conclusions

Supplementing BCAA along with other limiting AA to VLP diets more than recommended levels, partially recovered the reduced feed intake and body weight and serum IGF-I concentration, increased the plasma concentrations of BCAA and the mRNA abundance of hypothalamic *5HTR2A* and *NPY* and jejunal *5HTR7* and decreased the plasma serotonin. Thus, BCAA induced growth likely occurs through improved IGF-I and feed intake and changes in serotonin and its receptors, blood AA profile and hypothalamic NPY abundance. Our data provide evidence that both peripheral and central factors are associated with BCAA induced growth in pigs fed with protein restricted diets, but the role of these factors has yet to be assessed mechanistically. Further research is warranted to characterize the pathways by which BCAA regulate feed intake and growth.

## Author contributions

**A. Pezeshki** designed the study; **M. Habibi** performed the animal experiment; **M. Habibi** and **A. Pezeshki** wrote the manuscript with edits from other authors; **M. Habibi** drafted the manuscript, analyzed the data and created the graphs and tables; **C. Shili, J. Sutton** and **A. Pezeshki** contributed to performing the animal experiments; **P. Goodarzi** contributed to running the qPCR tests; **E. R. Maylem**, and **L. Spicer** performed the IGF-I assay; **A. Pezeshki** had the primary responsibility for the final content. All authors have read and approved the final manuscript.

## Conflict of interest

We declare that we have no financial and personal relationships with other people or organizations that might inappropriately influence our work, and there is no professional or other personal interest of any nature or kind in any product, service and/or company that could be construed as influencing the content of this paper.
