# Supplementary material for: Branched-chain amino acids partially recover the reduced growth of pigs fed with protein-restricted diets through both central and peripheral factors
Source: Anim Nutr. 2021 May 29;7(3):868–82. doi: 10.1016/j.aninu.2021.02.002 (PMC8484988; doi:10.1016/j.aninu.2021.02.002)
Supplement: Multimedia component 1 [file mmc1.docx]

# Branched-chain amino acids partially recover the reduced growth of pigs fed with protein-restricted diets through both central and peripheral factors

Mohammad Habibi ^a^, Cedrick Shili ^a^, Julia Sutton ^a^, Parniyan Goodarzi ^a^, Excel Rio Maylem ^a^, Leon Spicer ^a^, and Adel Pezeshki ^a,^*

^a^ Department of Animal and Food Sciences, Oklahoma State University, Stillwater, OK 74078, USA

^*^ Corresponding author.

E-mail address: [adel.pezeshki@okstate.edu](mailto:adel.pezeshki@okstate.edu) (A. Pezeshki)

# Appendices

**Appendix Table 1.** Chemical composition of supplemented dietary amino acids used for diet formulations

| **Chemical Composition** | **Amino acids^1^ (%), as-fed basis** | | | | | | | | **Amino acids^1^ (%), dry matter basis** | | | | | | | |
| --- | --- | --- | --- | --- | --- | --- | --- | --- | --- | --- | --- | --- | --- | --- | --- | --- |
|  | **Lys** | **Met** | **Thr** | **Trp** | **Val** | **Leu** | **Ile** | **Ala** | **Lys** | **Met** | **Thr** | **Trp** | **Val** | **Leu** | **Ile** | **Ala** |
| Dry matter, % | 92.3 | 99.6 | 99.5 | 99.5 | 100 | 100 | 100 | 100 | - | - | - | - | - | - | - | - |
| Crude protein, % | 74.9 | 58.5 | 73.2 | 85.3 | 74.7 | 66.7 | 67.1 | 98.2 | 81.2 | 58.7 | 73.6 | 85.7 | 74.7 | 66.7 | 67.1 | 98.2 |
| Crude fiber, % | < 1.0 | < 1.0 | < 1.0 | < 1.0 | < 1.0 | < 1.0 | < 1.0 | < 1.0 | < 1.1 | < 1.0 | < 1.0 | < 1.0 | < 1.0 | < 1.0 | < 1.0 | < 1.0 |
| Crude fat, % | 0.4 | < 0.2 | <0.2 | 0.2 | < 0.2 | < 0.2 | < 0.2 | < 0.2 | 0.4 | < 0.2 | < 0.2 | < 0.2 | < 0.2 | < 0.2 | < 0.2 | < 0.2 |
| Calcium, % | 0.04 | 0.02 | <0.01 | 0.02 | <0.01 | 0.02 | < 0.01 | < 0.01 | 0.04 | 0.02 | < 0.01 | 0.02 | < 0.01 | 0.02 | < 0.01 | < 0.01 |
| Phosphorus, % | 0.18 | < 0.01 | <0.01 | <0.01 | <0.01 | <0.01 | < 0.01 | < 0.01 | 0.2 | < 0.01 | < 0.01 | < 0.01 | < 0.01 | < 0.01 | < 0.01 | < 0.01 |
| ME^2^, Mcal/kg | 4.05 | 4.05 | 4.27 | 4.4 | 4.29 | 4.16 | 4.20 | 4.62 | 4.4 | 4.07 | 4.29 | 4.42 | 4.29 | 4.16 | 4.20 | 4.62 |

^1^ Lys: lysine, Met: methionine, Thr: threonine, Trp: tryptophan, Val: valine, Leu: leucine, Ile: isoleucine, Ala: alanine.

^2^ ME: metabolizable energy.

| **Appendix Table 2.** The sequences [forward (F) and reveres (R)], location on template, amplicon length (bp), and GenBank accession numbers for primers used for reverse transcription quantitative real-time polymerase chain reaction (RT-qPCR) ^1^ | | | | |
| --- | --- | --- | --- | --- |
| **Genes** | **Sequence (5’ → 3’)** | **Location on template** | **Amplicon length (bp)** | **GenBank accession no.** |
| Serotonin transporter (SERT) | **F:** TTTCCTTCTCTCGGTCATCG  **R:** GCATCCATTTCGGTGGTACT | 604-623  765-784 | 181 | XM_021067520.1 |
| Tryptophan hydroxylase 1 (TPH1) | **F:** TGGATCTGAACTGGATGCTG  **R:** CGGTTCCCCAGGTCTTAATC | 534-553  669-688 | 155 | XM_003122941.3 |
| 5-hydroxytryptamine-receptor 2B (5HTR2B) | **F:** CTCACGAGCTACAGCATTCATC  **R:** CCAGTGAGCCAAAGAGCATG | 687-708  837-856 | 170 | NM_001164019.1 |
| 5-hydroxytryptamine-receptor 7 (5HTR7) | **F:** GCAGATCAACTACGGCAGAG  **R:** CAGGTAGTTGGAGGGCTGAC | 222-241  344-363 | 142 | NM_214085.1 |
| 5-hydroxytryptamine-receptor 2A (5HTR2A) | **F:** GTTTCCTTGTCATGCCTGTG  **R:** GTTGAATCGCCTGTGATGG | 377-396  549-567 | 191 | NM_214217.1 |
| 5-hydroxytryptamine-receptor 1B (5HTR1B) | **F:** ACTACATTTACCAGGACTCCAT  **R:** CAGTGACCGTGTACATGGTGC | 110-131  317-337 | 228 | NM_214298.1 |
| Neuropeptide Y (NPY) | **F:** GCCAGATACTACTCGGCGTT  **R:** TGGGAACATTTTCCGTGCCT | 318-337  425-444 | 127 | NM_001256367.1 |
| Proopiomelanocortin (POMC) | **F:** TCCGAGAAGAGCCAGACG  **R:** GGCTTTGGGGTCGGCTTC | 804-821  912-929 | 126 | NM_213858.1 |
| Peptide YY (PYY) | **F:** GCAGAGGTATGGGAAACGTGA  **R:** GGGGGTGGTCACCATAGGTA | 248-268  348-367 | 120 | NM_001256528.1 |
| Glucagon gene (GCG) | **F:** AGAACTCCGCCGCAGACA  **R:** TAAAGTCTCGGGTGGCAAGATT | 420-437  481-502 | 83 | NM_214324.1 |
| Cholecystokinin (CCK) | **F:** CAGGCTCGAAAAGCACCTTC  **R:** GCGGGGTCTTCTAGGAGGTA | 260-279  397-416 | 157 | NM_214237.2 |
| Taste 1 receptor member 1 (T1R1) | **F:** TACAACGGTCTCCTCTCGGT  **R:** CAGCATGGCAAACACGTTGA | 3103-3122  3275-3294 | 192 | XM_013988748.2 |
| β-Actin | **F:** CTGCGGCATCCACGAAACT  **R:** AGGGCCGTGATCTCCTTCTG | 944-962  1071-1090 | 147 | XM_003124280.5 |

^1^Primers were obtained from previously published studies: SERT (Smith et al., 2014), TPH1 (Cremer et al., 2015), 5HTR2B (Cremer et al., 2015), 5HTR7 (Nakamura et al., 2008), 5HTR2A (Cremer et al., 2015), 5HTR1B (Cremer et al., 2015), NPY (Tian et al., 2019), POMC (Tian et al., 2019), PYY (Tian et al., 2019), GCG (Tian et al., 2019), CCK (Tian et al., 2019), T1R1 (Tian et al., 2019) and β-Actin (Yin et al., 2015)

| **Appendix Table 3.** The host, dilution and supplier of primary and secondary antibodies for immunoblotting. | | | |
| --- | --- | --- | --- |
| **Antibodies** | **Host** | **Dilution** | **Vendor** |
| Anti- MC4-R^1^ | Rabbit | 1:400 | Abcam, Cambridge, MA, #ab24233 |
| Anti- TPH2^1^ | Goat | 1:500 | Abcam, Cambridge, MA, #ab121013 |
| Anti- FGF21^1^ | Rabbit | 1:400 | Santa Cruz Biotechnology, Inc., USA, #SC-292879 |
| Anti-GAPDH | Mouse | 1:1000 | Abcam, Cambridge, MA, #ab105428 |
| Anti-Goat IgG H&L (HRP) | Donkey | 1:2000 | Abcam, Cambridge, MA, #ab205723 |
| Anti-Rabbit IgG H&L (HRP) | Goat | 1:1600 | Abcam, Cambridge, MA, #ab205718 |
| ^1^ MC4R: Melanocortin-4-receptor, TPH2: Tryptophan hydroxylase 2, FGF21: Fibroblast growth factor 21. | | | |


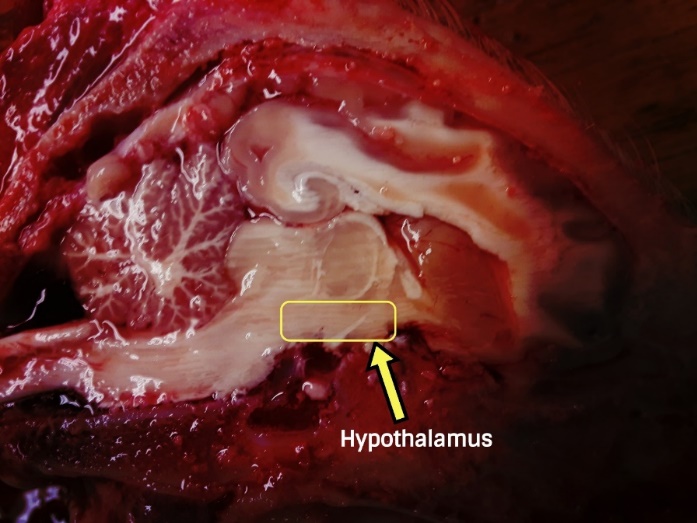


**Appendix Fig. 1.** Midsagittal view of the pig brain.

Immediately after euthanasia, the pigs were decapitated and the hypothalamus was extracted following the midsagittal dissection of the head which divided the right and left sides of the brain into two equal parts. Then, extracted samples were snap-frozen in liquid nitrogen and stored at -80 °C until further analysis.

**
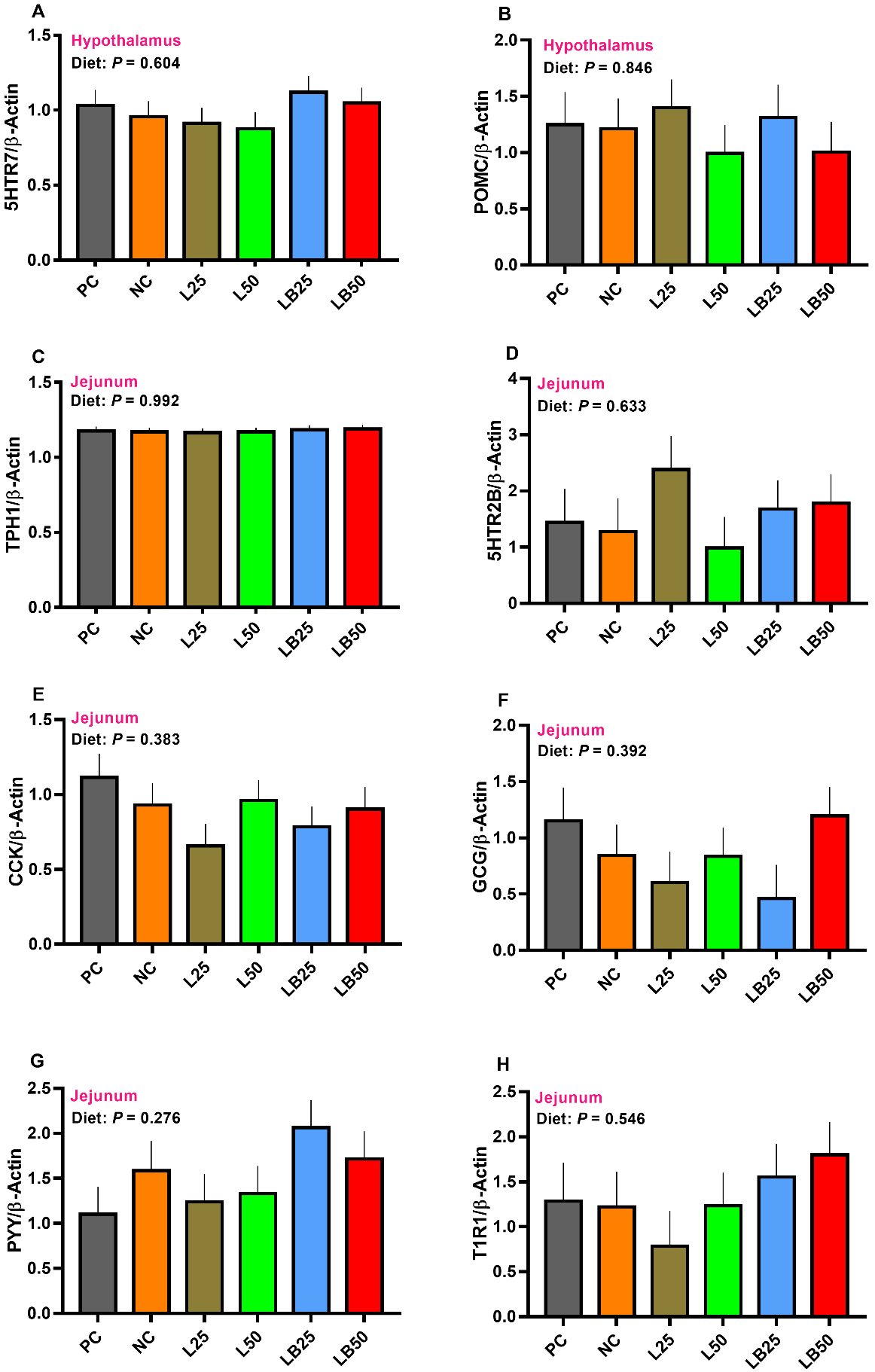
**

**Appendix Fig. 2.** The effect of very low protein diet supplemented with combination of limiting and branched-chain amino acids on mRNA abundance of feed intake markers in hypothalamus and jejunum

(**A**) 5-hydroxytryptamine-receptor 7 (5HTR7), (**B**) proopiomelanocortin (POMC), (**C**) tryptophan hydroxylase 1 (TPH1), (**D**) 5-hydroxytryptamine-receptor 2B (5HTR2B), (**E**) cholecystokinin (CCK), (**F**) glucagon gene (GCG), (**G**) peptide YY (PYY), (**H**) taste 1 receptor member 1 (T1R1). PC (positive control), standard protein diet; NC (negative control), low protein diet; L25, low protein diet with supplemented limiting amino acids (LAA, *i.e.* Lys, Met, Thr and Trp) 25% more than NRC (NRC 2012) requirements; L50: low protein diet with supplemented LAA 50% more than NRC requirements; LB25: low protein diets with supplemented LAA and branched-chain amino acids (BCAA, *i.e.* Leu, Ile and Val) 25% more than NRC requirements; LB50: low protein diet with supplemented LAA and BCAA 50% more than NRC requirements. The relative mRNA abundance was determined by qPCR with using β-Actin as a reference target. The values are means ± SEM, *n*=8.
